# Early Deformation of Deep Brain Stimulation Electrodes Following Surgical Implantation: Intracranial, Brain, and Electrode Mechanics

**DOI:** 10.3389/fbioe.2021.657875

**Published:** 2021-06-11

**Authors:** Frédéric Chapelle, Lucie Manciet, Bruno Pereira, Anna Sontheimer, Jérôme Coste, Youssef El Ouadih, Ruxandra Cimpeanu, Dimitri Gouot, Yuri Lapusta, Béatrice Claise, Valérie Sautou, Yassine Bouattour, Ana Marques, Adrien Wohrer, Jean-Jacques Lemaire

**Affiliations:** ^1^Sigma Clermont, Clermont Auvergne Institut National Polytechnique, Clermont-Ferrand, France; ^2^Université Clermont Auvergne, Centre National de la Recherche Scientifique, Clermont Auvergne Institut National Polytechnique, Institut Pascal, Clermont-Ferrand, France; ^3^Direction de la Recherche Clinique et de l’Innovation, Centre Hospitalier Universitaire de Clermont-Ferrand, Clermont-Ferrand, France; ^4^Service de Neurochirurgie, Centre Hospitalier Universitaire de Clermont-Ferrand, Clermont-Ferrand, France; ^5^Service de radiologie, Centre Hospitalier Universitaire de Clermont-Ferrand, Clermont-Ferrand, France; ^6^Centre National de la Recherche Scientifique, Clermont Auvergne Institut National Polytechnique, Institut de Chimie de Clermont-Ferrand, Université Clermont Auvergne, Clermont-Ferrand, France; ^7^Service de neurologie, Centre Hospitalier Universitaire de Clermont-Ferrand, Clermont-Ferrand, France

**Keywords:** DBS, electrode, deformation, biomechanics, brain

## Abstract

**Introduction:**

Although deep brain stimulation is nowadays performed worldwide, the biomechanical aspects of electrode implantation received little attention, mainly as physicians focused on the medical aspects, such as the optimal indication of the surgical procedure, the positive and adverse effects, and the long-term follow-up. We aimed to describe electrode deformations and brain shift immediately after implantation, as it may highlight our comprehension of intracranial and intracerebral mechanics.

**Materials and Methods:**

Sixty electrodes of 30 patients suffering from severe symptoms of Parkinson’s disease and essential tremor were studied. They consisted of 30 non-directional electrodes and 30 directional electrodes, implanted 42 times in the subthalamus and 18 times in the ventrolateral thalamus. We computed the x (transversal), y (anteroposterior), z (depth), torsion, and curvature deformations, along the electrodes from the entrance point in the braincase. The electrodes were modelized from the immediate postoperative CT scan using automatic voxel thresholding segmentation, manual subtraction of artifacts, and automatic skeletonization. The deformation parameters were computed from the curve of electrodes using a third-order polynomial regression. We studied these deformations according to the type of electrodes, the clinical parameters, the surgical-related accuracy, the brain shift, the hemisphere and three tissue layers, the gyration layer, the white matter stem layer, and the deep brain layer (type I error set at 5%).

**Results:**

We found that the implanted first hemisphere coupled to the brain shift and the stiffness of the type of electrode impacted on the electrode deformations. The deformations were also different according to the tissue layers, to the electrode type, and to the first-hemisphere-brain-shift effect.

**Conclusion:**

Our findings provide information on the intracranial and brain biomechanics and should help further developments on intracerebral electrode design and surgical issues.

## Introduction

Deep brain stimulation (DBS) is a functional treatment proposed to patients suffering from severe neurological diseases, such as Parkinson’s disease, essential tremor, and dystonia (Fox et al., 2018; Ferreira et al., 2019). DBS has been shown to dramatically improve tremor, akinetic-rigid syndrome, dystonic movements, and dyskinesia, using electrodes that deliver chronic biphasic electric stimulation at a frequency usually inferior to 200 Hz and a low voltage of about 3 Volts. While the medical outcomes are well known, the mechanical aspects of the implantation process have received little attention.

**FIGURE 1:**
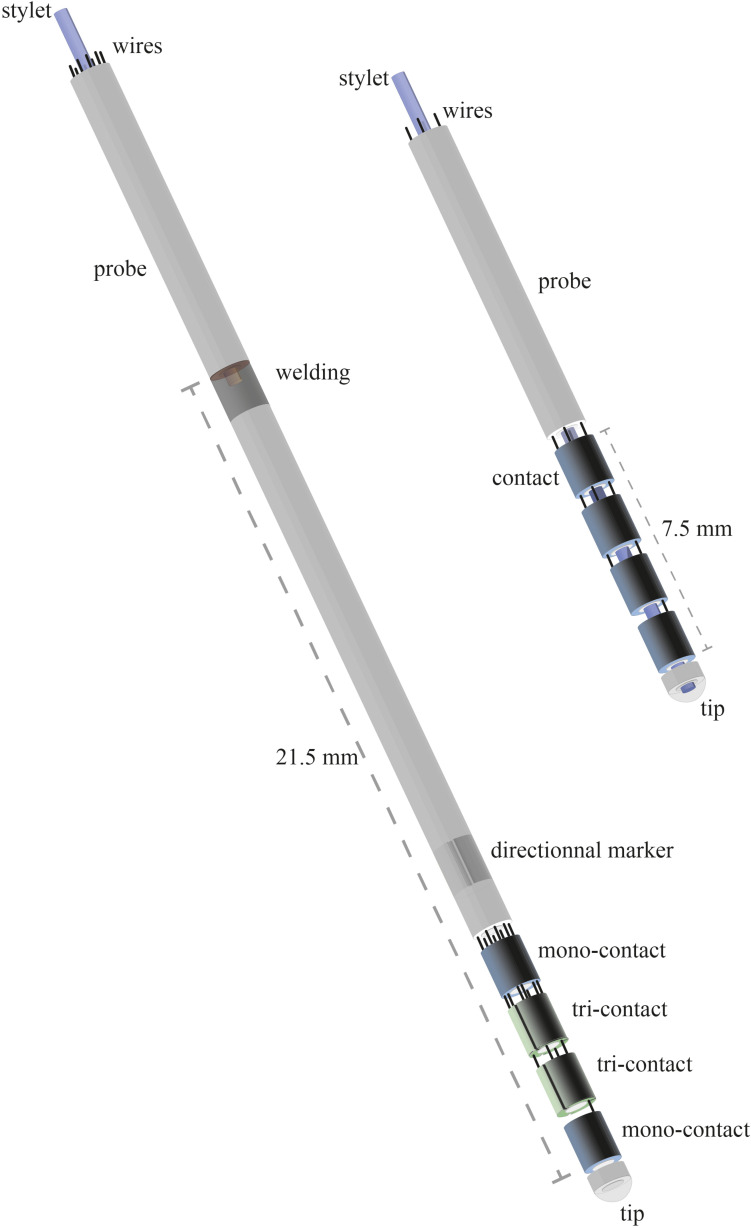
Electrodes of deep brain stimulation. Directional (left) and non-directional (right) electrodes, with rigid distal parts measuring, respectively, 21.5 and 7.5 mm (see text methods for details).

**FIGURE 2:**
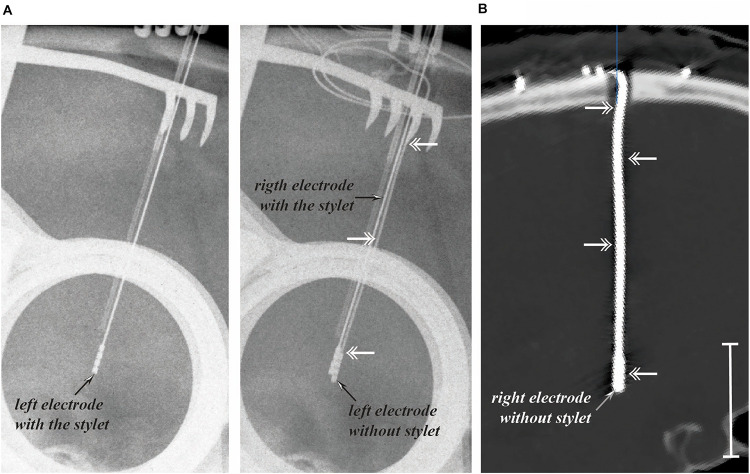
Electrode deformation following stylet retraction. Intraoperative X-rays (**A**; lateral projection; anterior is on the right) showing the electrode deformation (double arrows) after removal of stylet (left and intermediate rows) and immediately after removal of the stereotactic frame on postoperative CT scan (**B**; pseudo sagittal reconstructed slice along the right electrode; anterior is on the right; white vertical bar = 25 mm).

A standard DBS electrode is a flexible polymer tube with a semirigid retractable metallic stylet inside, enabling to push the electrode toward the deep-seated target. Thus, the DBS electrode is also a lead, in which several electrodes, usually four, i.e., the contacts, are crimped distally at the surface; the conductor wires are embedded in the tube wall (Figure 1). When the position of the contacts relative to the intended or selected anatomo-functional target is reached, the stylet is removed, and the lead is fixed to the cranial vault, secured by screws. For convenience, the word electrode will be used in the following pages instead of DBS electrode or DBS lead. Following the implantation of electrodes within the right and/or left hemispheres, most surgical teams perform a CT scan after removal of the stereotactic frame, seeking hemorrhagic complications and checking electrode positioning, with or without coregistration with preoperative magnetic resonance imaging (MRI) data. From intraoperative X-rays and early postoperative image data sets, the deformation undergone by the electrodes is visible (Figure 2).

The observed deformation raises several questions. Some authors focus on the difference between the intended-to-target point and the observed location of the contacts and calculate an “error of targeting” (Li et al., 2016), yet doing so they skip the deformation of the electrode and hide the different causes of the mismatch. However, although reductive, it emphasizes the notion of surgical inaccuracy, which is true for several reasons: (1) current stereotactic frames (surgical instrument for the implantation of the electrodes) and robotized systems have an intrinsic mean geometrical error usually of 1 mm (von Langsdorff et al., 2015; Alptekin et al., 2019); (2) manual measurements on X-ray films and surgical software introduce errors or inaccuracy, such as the difficulties of reading stereotactic rules (visual interpolation) and contact position on X-rays; (3) manipulation of surgical tools introduces limitations to the leaving and securing of electrodes in place (Contarino et al., 2013), with a global (2 + 3) precision roughly estimated, at around 1 mm; (4) erroneous targeting should be exceptional (pure error of targeting); and (5) mechanical issues can be encountered during the progression of the electrode, such as friction at the dura aperture, deviation by a foreign body along the tract such as surgical wax used for bone hemostasis, presence of significant blood vessels and arachnoid adherences, and changing of medium from tissue to ventricle and conversely. This notion of surgical inaccuracy should be separate from the delayed lead migration explained by mechanical issues such as technical error, repetitive dystonic head movement, and Twiddler’s syndrome and reported between two CT scans separated by a mean interval of 1 year (differences of tip position or length of electrode > 3 mm) (Morishita et al., 2017).

Others have focused their attention on the deformation as a result of the brain shift (Bentley et al., 2017; Darbin et al., 2018), i.e., the distortion and moving of the brain within the braincase following modifications of intracranial gradients of pressure. This happens notably between two osteo-dural compartments such as the supratentorial and infratentorial fossa and the right and left cavities of the brain hemispheres. The main cause of brain shift is cerebrospinal fluid (CSF) leakage, following the opening of the dura, and accompanied by air penetration. Intraoperatively, the brain shift can also be the witness of a worrying but rare significant hemorrhagic complication. The CSF leakage is mainly caused by a large dura opening that facilitates the leakage and a non-recumbent position that facilitates the air penetration because of the decrease in the intracranial pressure. Minimizing the CSF leakage dramatically reduces the brain shift and its consequences (Nazzaro et al., 2010; Petersen et al., 2010; Slotty et al., 2012). One should bear in mind that the deep brain is less sensitive to the shift. Indeed, it is well-known in stereotactic neurosurgery that the deep brain is somewhat relatively fixed because of the bracing by large vessels and cranial nerves; this is one of the historical rationales on which the stereotactic neurosurgery relies. The brain shift can also participate to the surgery-related errors (Sillay et al., 2013). Moreover, it is potentially more impactful for electrode placement within the fronto-basal region (Choi et al., 2018).

The electrode deformation specifically has received little interest. It seems, however, that it is maximum during the 2 weeks following the implantation and more pronounced in case of pallidal versus subthalamic placements (Lalys et al., 2014).

Our goal was to analyze the observable deformations of electrodes and the brain immediately after electrode implantation. We hypothesized that these deformations, visible as from the removal of stylet, and documented in 3D by CT scan about an hour after implantation, could be, at least partially, the witness of the intrinsic mechanical behavior of the brain following electrode positioning, and before the formation of a potential brain edema. The electrode would play the role of an active probe, which introduced its own mechanical constraints, revealing local mechanical phenomena. For this purpose, electrode deformation, brain shift, and surgical inaccuracy were studied in a series of 30 consecutive patients operated on bilaterally and using two kinds of electrodes: a historical, non-directional, electrode, where the current is delivered circumferentially by each cylindric contact, and a recent model, directional, where the current can be delivered in one, two, or three different directions by contacts occupying only a third of the cylindrical section of the electrode.

## Materials, Methods, and Theory

### Material

**FIGURE 3:**
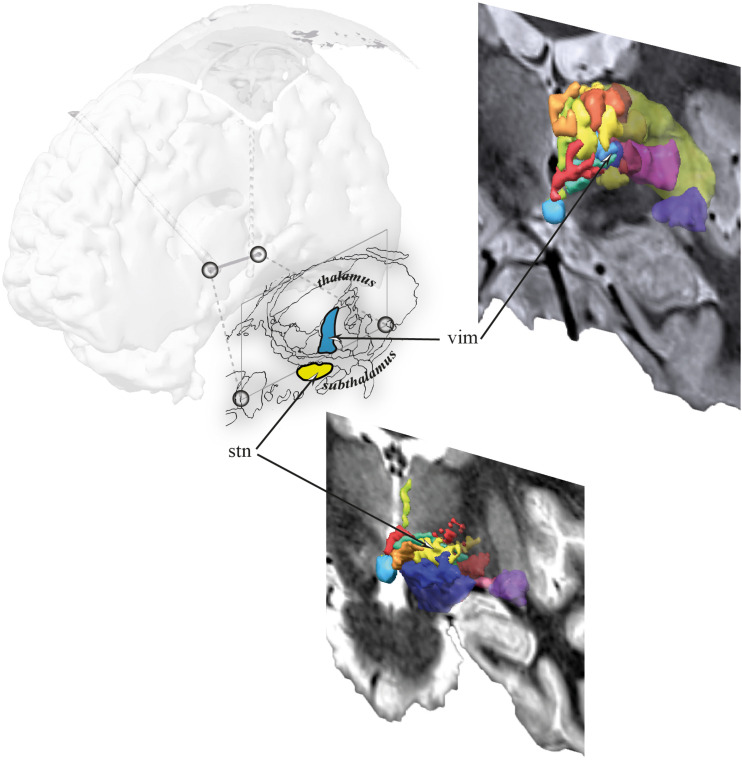
Location of thalamic and subthalamic electrodes. Sagittal section 13.5 mm lateral to the midline plan (dark lines) and coronal MRI slices, going through the ventral intermediate nucleus (vim, blue; ventrolateral thalamus), and the area of subthalamic nucleus (stn, yellow; subthalamus).

Sixty electrodes of 30 patients (bilateral implantations) were studied in a retrospective observational monocentric study (*Commission Nationale de l’Informatique et des Libertés*, CNIL, agreement M200702). These electrodes were implanted in the ventrolateral thalamus (nucleus ventral intermediate) and the immediate subthalamus (subthalamic nucleus and its immediate neighborhood) (Figure 3). The first electrode was systematically implanted in the left hemisphere. The clinical material consisted of 42 subthalamic electrodes (21 Parkinson’s disease) and 18 ventrolateral thalamic electrodes (eight essential-tremor patients and 1 Parkinsonian). Thirty non-directional electrodes (DBS 3389; Medtronic; Minneapolis, MN, United States) and 30 directional electrodes (Infinity DBS; Abbott Neuromodulation; Plano, TX, United States) were implanted, respectively, 20 times within the subthalamus and 10 times within the ventrolateral thalamus, and 22 times within the subthalamus and eight times within the ventrolateral thalamus. The mean age of the patients (*n* = 30) was 62 ± 8.1 years [41;78] (Table 1). The two types of electrodes were implanted consecutively, following the regular recruitment of patients (current institutional protocol of DBS surgery according to the national medical guidelines; Haute Autorité en Santé). The non-directional electrodes were implanted first, followed by the directional electrodes, which were recently authorized (Food and Drug Administration and European Union). Each patient received a unique neuropacemaker for the two electrodes, which were of the same type, non-directional or directional, in the right and left hemispheres.

**TABLE 1:** Clinical material.

Data		Electrode type	
		
		Non-directional (*n* = 30)	Directional (*n* = 30)
Age (years, mean ± sd)		62.7 ± 8.67	61.2 ± 7.73
Gender (n): female/male		7/8	11/4
disease_target (n):		10/5	11/4
MPI_subthalamus/Tremor_Vim			
Hemisphere size (cm^3^, mean ± sd)	Right	487.9 ± 43.4	473.0 ± 61.9
	Left	483.5 ± 42.4	464.4 ± 66.1
Brain shift index (% × 10^–4^, mean ± sd)	Right	1.19 ± 4.44	1.69 ± 4.81
	Left	11.77 ± 37.29	0.18 ± 0.65
Gyration-WM frontier (mm, mean ± sd)	Right	24.5 ± 3.6	26.7 ± 4.7
	Left	25 ± 2.6	25 ± 3.7
WM-DB frontier (mm, mean ± sd)	Right	39.6 ± 3.65	40.6 ± 3.42
	Left	40.8 ± 2.5	41.6 ± 3.9
Surgery-related error (Euclidian distance, mm, mean ± sd)	Right	0.92 ± 0.5	1.15 ± 0.5
	Left *	1.06 ± 0.5	1.5 ± 0.5
x deformation (mm, mean ± sd)	Right	0.18 ± 0.20	0.12 ± 0.16
	Left *	0.14 ± 0.23	−0.13 ± 0.13
y deformation (mm, mean ± sd)	Right *	0.06 ± 0.20	0.33 ± 0.25
	Left *	0.12 ± 0.43	0.04 ± 0.23
z deformation (mm, mean ± sd)	Right *	−0.14 ± 0.12	0.07 ± 0.17
	Left *	−0.09 ± 0.22	0.06 ± 0.12
Torsion (mm^–1^, mean ± sd)	Right	0.015 ± 0.056	−0.007 ± 0.044
	Left	−0.016 ± 0.076	0.025 ± 0.044
Curvature (mm^–1^, mean ± sd)	Right	0.0029 ± 0.0012	0.0025 ± 0.001
	Left *	0.0036 ± 0.0027	0.0022 ± 0.007

Comparison between the non-directional and directional electrodes.

*Statistically significant, p ≤ 0.05.

*MPI, Parkinson’s disease; tremor, essential tremor; WM, white matter stem; DB, deep brain.*

The 30 non-directional electrodes were made of polyurethane 80A polymer (DBS 3389; Medtronic; Minneapolis, MN, United States). The stylet (tungsten) was insulated with parylene; the four contacts were made of platinum–iridium, and the conductor wires were insulated with fluoropolymer. The stylet went to the distal tip of the electrode; each contact measured 1.5 mm length and was separated by 0.5 mm. The rigid distal part measured 7.5 mm. The 30 directional electrodes were made of 55D Bionate^®^ thermoplastic polycarbonate urethane (Infinity DBS; Abbott Neuromodulation; Plano, TX, United States). The four distal contacts were made of platinum–iridium. Each contact of 1.5-mm length was separated by 0.5 mm. The two intermediate contacts split into three thirds-of-cylinder facets (directional contacts). Thus, the electrode had eight contacts (two non-directional contacts and two tri-directional contacts). The stylet goes to the welding zone of wires, 21.5 mm above the distal tip, hence the rigid distal part measured 21.5 mm. The non-directional and directional electrodes are schematized in Figure 1.

The measurements of the electrode parameters were realized from the immediate postoperative CT scan (LightSpeed, GE, United States) image data set: 230 axial slices (dual energy); matrix 512^2^; pixel size 0.488 × 0.488 mm^2^; and thickness 0.625 mm. After coregistration of pre- and postoperative imaging data sets (mutual information coefficient algorithm; iPlan 3.0; Brainlab, Germany), the measurements relative to the brain structures were realized from the preoperative morphologic MRI (Avanto, 1.5 T; Siemens, Germany): white matter attenuated inversion-recovery sequence (Zerroug et al., 2016), acquisition in the coronal plane, matrix 512^2^; and thickness 2 mm.

### Methods and Theory

**FIGURE 4:**
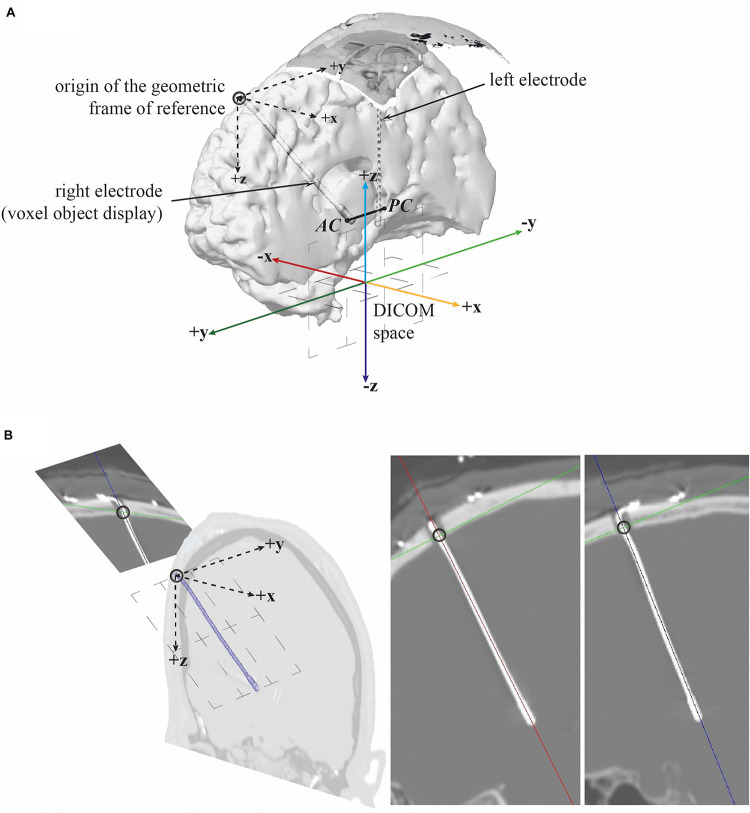
Geometrical frame of reference. **(A)** Antero-lateral left view of the right hemisphere showing the electrode and the geometrical frame of reference [the anterior commissure-posterior commissure (ACPC) line is given for information]. **(B)** Reconstructed CT-scan slice, pseudo sagittal, pseudo coronal, and pseudo sagittal (zoom in, from left to right) along the right electrode showing the proximal point (black circle) that served as origin of the geometric frame of reference.

We studied the intracranial part of electrodes, from the proximal point where the electrode goes through the cranial burr hole to the distal deep-seated extremity. This proximal point served as origin for the calculation of the length of electrode within the hemisphere and the deformation parameters and as the origin of the geometric frame of reference; all Digital Imaging and Communications in Medicine (DICOM)^®^ coordinates were converted accordingly. The geometrical frame of reference is displayed in Figure 4. Anatomic-landmark referencing or reorientation (e.g., ACPC, anterior commissure–posterior commissure, or MNI, Montreal Institute of Technology) was not used because the electrodes served as landmarks and the derived parameters were normalized proportionally by individuals for further analysis.

No electrode crossed the ventricles and the sulci (double obliquity trajectory, entrance point at the surface of the 2nd frontal gyrus; 3D planning; iPlan 3.0, Brainlab, Germany). The left and right electrodes of each patient were extracted as follows (Thermo ScientificTM AmiraTM, v 6.4, Hillsboro, OR, United States): automatic segmentation of voxels using signal thresholding; manual removal of bony structures and electrode artifacts; manual specification of the proximal point at the level of the internal layer of cortical bone (triplanar drawing tools); and automatic computation of the skeleton of the electrode. We calculated the length of the electrode within the hemisphere (Euclidian distance, mm, between the proximal and distal points), the percentage of the electrode length crossing the extracerebral space (between the proximal point and the surface of the cortex), and the percentage of the distal rigid part (7.5 mm, non-directional; 21.5 mm, directional).

**FIGURE 5:**
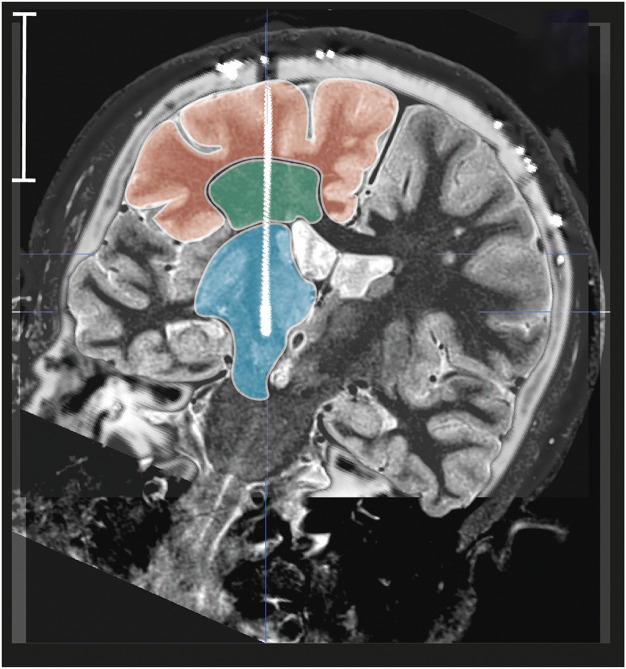
Tissue layers. Example of overlay of postoperative CT scan and preoperative MRI (reconstructed pseudo coronal slice along the right electrode) showing the three tissue layers: the superficial gyration layer (red), the intermediate white-matter layer (green), and the deep brain layer (blue); white vertical bar = 50 mm.

We defined three layers of brain tissue that could have specific mechanical properties (Lalys et al., 2014; Budday et al., 2017). These successive layers were systematically crossed by the electrodes: the superficial gyration layer, the intermediate white-matter stem (WMS) layer, and the deep brain layer (Figure 5). The layers were directly identified on the preoperative MRI. Following the co-registration, we measured (in mm) the distance between the proximal point and the superficial and deep frontiers, respectively, between the gyration and the white matter stem layers and between the white matter stem and the deep brain layers. The location of the tissue frontiers was used for further analysis of the electrode deformation.

**FIGURE 6:**
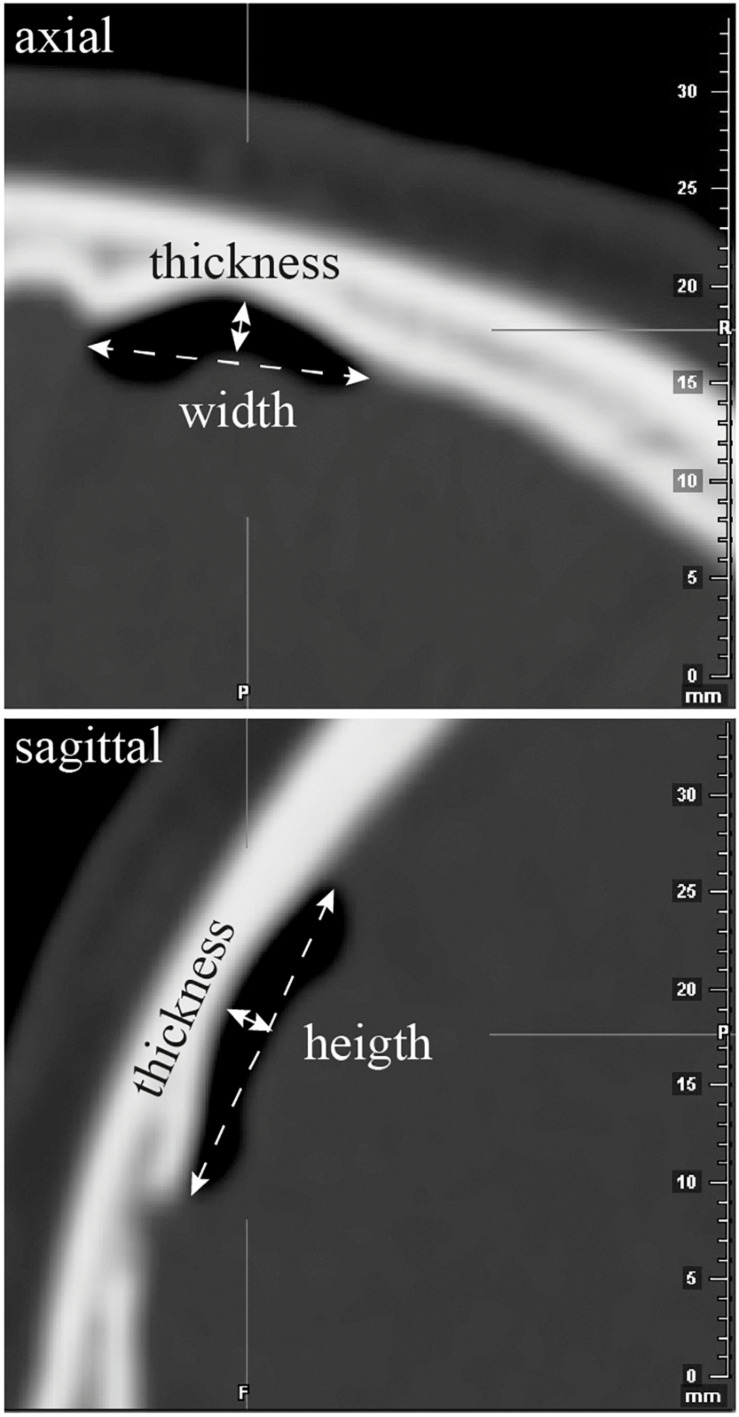
Air bubble visible against the frontal pole on postoperative CT scan. Principle of the measurements of the width, thickness, and height of the bubble air on axial and sagittal CT-scan slices.

The brain shift was approximated, for each hemisphere of the 30 patients, as follows: we measured (in mm) the height, width, and thickness of the air bubble visible against the frontal pole on the postoperative CT scan (patient in the recumbent position all along the surgical procedure and the CT scan acquisition; no electrode tracks going through the ventricles) (Figure 6); we roughly estimated the volume of the air collection (in mm^3^; overestimation); we computed the volume of the hemisphere (in mm^3^; iPlan 3.0; Brainlab; Germany); and we calculated the ratio of volumes of air collection/hemisphere, which was named the brain shift index (the higher index value, the bigger brain shift).

**FIGURE 7:**
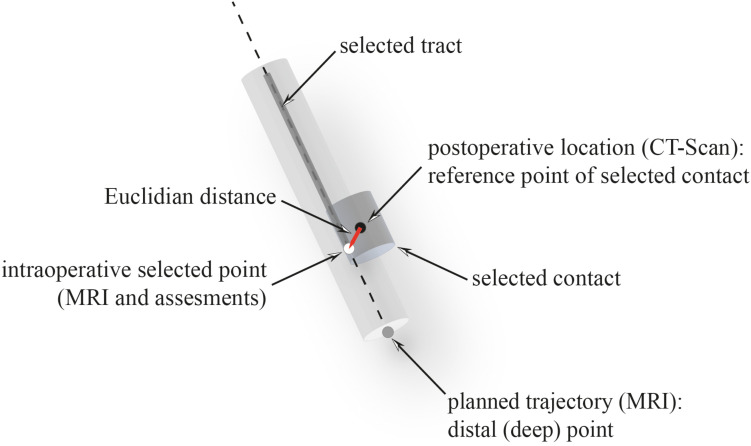
Surgical-related inaccuracy. The Euclidian distance (red) was calculated between the reference point (black) of the selected contact and the selected point (white) along the selected tract.

The surgical-related inaccuracy was specified as the Euclidean distance, measured between the optimal point selected intraoperatively along the planned trajectory and according to the MRI anatomy and/or the clinical or electrophysiological assessments (passive or active), and the selected contact point (distal, proximal, or midpoint of a contact) of the electrode (iPlan 3.0; Brainlab, Germany) (Figure 7). This electrode contact point was extrapolated from the postoperative CT-scan artifact (Hemm et al., 2009). Following our usual surgical procedure, we systematically used intraoperatively two exploratory tracts by hemisphere: the central tract, along the planned trajectory, and the posterior or lateral or posterolateral tract distant of 2 mm (radius). Because of the 3D measurement, all other things being equal, the surgical-related distance of inaccuracy could have been longer than 2 mm. Practically, the Euclidian distance was measured on the postoperative CT scan where both the electrodes and the trajectories were displayed after coregistration of pre- and postoperative image datasets (iPlan 3.0; Brainlab; Germany; geometric resolution = 0.1 mm).

**FIGURE 8:**
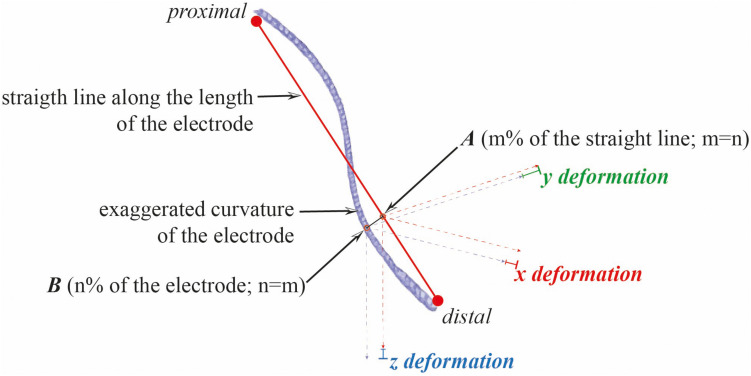
Deformation of electrodes. The deformation of each electrode was computed in the x, y, and z directions by calculation of the distance between the skeleton of the electrode and the straight line along its length, normalized to 100 points.

**FIGURE 9:**
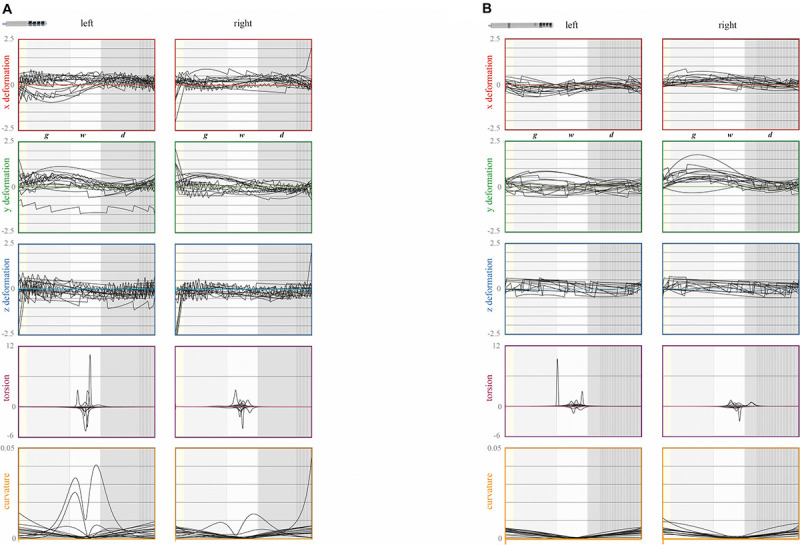
Deformation parameters of the electrode models. (**A**, non-directional, *n* = 30; **B**, directional, *n* = 30; 15 electrodes per hemisphere, right and left), from top to bottom: x (red), y (green), and z (blue) deformation values (from –2.5 to 2.5 mm) of the skeleton (see text for details); torsion (purple; from –6 to 12 mm^–1^) and curvature (orange; from 0 to 0.05 mm^–1^) values along the electrodes; abscissa, depth along the electrode crossing the subdural space (yellow), the gyration layer (light gray), the white matter stem (pale gray), and the deep brain (dark gray) (proportional display); the distal rigid part of electrodes is overlaid (vertical hatching).

The curve of each electrode was modeled from the skeleton using a third-order polynomial regression (Lalys et al., 2014), leading to an acceptable approximation of the electrode with a median *R*^2^-value of 0.98 (monodirectional electrodes, 0.969; multidirectional electrodes, 0.981) and a median root mean square value of 0.29 mm (monodirectional electrodes, 0.35 mm; multidirectional electrodes, 0.22 mm). The aspects of the polynomial regressions represented fairly the observed deformation on X-rays and CT scans. Deformation parameters were computed for each electrode: the x, y, and z deformation (mm), i.e., the distances between the curve and the straight line along the length of the skeleton (Figure 8), and the torsion (mm^–1^) and the curvature (mm^–1^) values; coarsely, the higher the deformations, torsions, and curvatures, the higher the values (Frenet–Serret formula; Python library). The electrode (skeleton, polynomial function and straight line) was normalized with a 100-point function. The representation of the deformation parameters of all electrodes is shown in Figure 9.

The positive values of the deformation, along the x-axis, y-axis, and z-axis, meant that the electrode bent, respectively, toward the midline, posteriorly, and inferiorly (Figure 4). We computed the regional values of the deformation parameters, i.e., by tissue layers (gyration, WMS, and deep brain): the average value and the intraclass correlation coefficient (ICC) of the deformation parameters (x, y, z, torsion, and curvature). These regional parameters were compared between pairs of tissue layers, gyration versus WMS, gyration versus deep brain, and WMS versus deep brain, and between the right and left hemispheres. We also compared the regional parameters of the two types of electrode in the deep brain, where the rigid distal part was positioned, and for the right and left hemispheres. The deformation parameters were also analyzed according to the type of electrodes, the hemisphere, the brain shift index, and the surgical-related inaccuracy.

Statistical analyses were performed using Stata software, Version 15 (StataCorp, United States). Continuous data were expressed as mean and standard deviation according to statistical distribution. The assumption of normality was assessed using the Shapiro–Wilk test. The comparisons between independent groups (type of electrode) were carried out, for continuous variables, using Student’s *t*-test or Mann–Whitney test when the requirements of *t*-test were not met. The quality variance assumption was studied by using the Fisher–Snedecor test. For the categorical data (i.e., gender, disease), chi-squared or Fisher’s exact tests were performed. For correlated data (when several measures were collected for the same patient, i.e., by hemisphere and tissue layer), random-effect models (i.e., mixed linear regression) were used to evaluate the following effects: type of electrode, hemisphere, tissue layer and their interactions, and subject (as random effect). These models were suitable to model between- and within-subject variability expressed as ICC. Like most correlation coefficients, the ICC ranges from 0 to 1. A high ICC value, close to 1, indicates high similarity between values from the same patient, whereas a low ICC, close to 0, means that values from the same patient are not correlated. More precisely, ICC values were interpreted according to thresholds (Altman, 1990) as < 0.6, low; 0.6–0.8, moderate; and > 0.8, high. The normality of residuals from these models was studied as aforementioned. When appropriate, a logarithmic transformation has been applied. The tests were two-sided with a type I error set at 5%. A Sidak type I error correction was applied for multiple comparisons.

## Results

### Clinical Material

The two series of patients with the 30 non-directional electrodes and the 30 directional electrodes were comparable according to the age (*t*-test; *p* = 0.6132), to the gender (Pearson Chi^2^; *p* = 0.136), to the diseases (Pearson Chi^2^, *p* = 0.690), and to the brain shift index (Kruskal–Wallis; right, *p* = 0.9669; left, *p* = 0.1585; both, *p* = 0.3297) (Table 1). The mean volumes of the right, left, and both hemispheres were, respectively, 480.5 ± 53 cm^3^, 474 ± 55 cm^3^, and 954.4 ± 106 cm^3^. They were comparable according to the type of electrodes (Kruskal–Wallis; right, *p* = 0.3095; left, *p* = 0.3095; both, *p* = 0.3297) (Table 1).

However, the brain shift index was substantially higher on the left and for the non-directional electrodes, respectively, on the left 11.77 ± 37.29% × 10^–4^ for the non-directional versus 0.18 ± 0.65% × 10^–4^ for the directional, and 1.19 ± 4.44 versus 1.69 ± 4.81 on the right, although this did not reach statistical significance because of the variability (right versus left, Wilcoxon, *p* = 0.07; left, non-directional versus directional, Kruskal–Wallis, *p* = 0.07). The average dimensions of the intracranial frontal air bubbles, thickness, width, and height were small, and respectively, on the left, 1.54 ± 2.86 mm, 1.91 ± 6.84 mm, 3.20 ± 7.56 mm, and on the right, 0.51 ± 1.34 mm, 1.28 ± 3.72 mm, 1.63 ± 4.62 mm.

The position of tissue frontiers was comparable in the two series of patient electrodes (*t*-test; right, gyration-WM, *p* = 0.1595, WM-DB, *p* = 0.4722; left, gyration-WM, *p* = 0.9865, WM-DB, *p* = 0.4846) (Table 1).

The surgical-related inaccuracy was higher on average on the left, respectively, 1.28 ± 0.5 mm (*n* = 30, left) and 1.03 ± 0.5 mm (*n* = 30, right) (Wilcoxon, *p* = 0.0427). There was no difference in mean Euclidian distance, on the right according to the type of electrode, but on the left hemisphere the surgery-related inaccuracy was superior for the directional electrodes (*t*-test; R, *p* = 0.1935; L, *p* = 0.01) (Table 1).

### Electrodes

The average length of electrodes within the right and left hemispheres was 67.7 ± 3.6 mm [58.9; 76.1]. This length was longer, by anatomic configuration, for the subthalamic electrodes than for the ventrolateral–thalamus electrodes, respectively 68.1 ± 9.1 mm [62.5; 76.1] and 64.5 ± 3.2 mm [58.9; 70.4]. The percentage of the electrode length, which was rigid, by technology, at the distal extremity, was on average, respectively, 11.1 ± 0.6% [10; 13] for the non-directional electrodes and 31.77 ± 1.7% [29; 36] for the directional electrodes. The length of the extracerebral segment of the electrode (*n* = 60) was on average 4.12 ± 0.8 mm [2.6, 7.2], which represented 6 ± 1.2% [3.7, 10.6] of the electrode length.

The mean electrode deformation was different according to the type of electrode and the direction, along y and z in both hemispheres and along x in the left hemisphere (Kruskal–Wallis; x right, *p* = 0.2372; y right, *p* = 0.0054; z right, *p* = 0.002; x left, *p* = 0.003; y left, *p* = 0.04; z left, *p* = 0.014): x deformation in the left hemisphere was toward the midline for the non-directional electrodes, and opposite, toward the lateral part of the hemisphere for the directional electrodes; y deformation was posterior and higher, in the left hemisphere for the non-directional electrodes and in the right hemisphere for the directional electrodes; and z deformation was higher toward the depth in both hemispheres for the non-directional electrodes. The mean torsion was not different according to the type of electrode (Kruskal–Wallis; right, *p* = 0.5476; left, *p* = 0.1647), but the mean curvature of the non-directional electrodes was higher in the left hemisphere (Kruskal–Wallis; right, *p* = 0.3725; left, *p* = 0.049) (Table 1).

**FIGURE 10:**
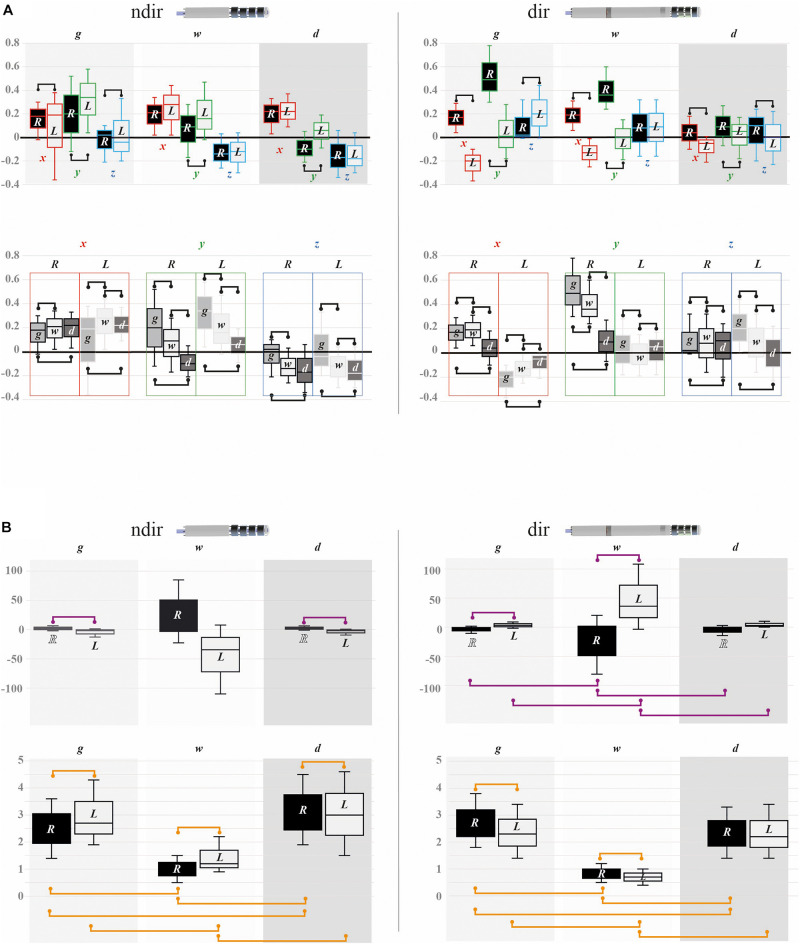
Deformation parameters. Values of the deformation parameters of the curves of non-directional (ndir = 30) and directional (dir = 30) electrodes (15 per hemisphere): box plot; median value = central line; median interquartile range, IRQ25–IRQ75 = extremities of the segment; the significant differences are represented by handles (random-effect model, *p* < 0.05; see Supplementary Table 1 for values). **(A)** x (red), y (green), and z (blue) deformation values (–0.4 to 0.8 mm) according to the three tissue layers (gyration, g; white matter stem, w; deep brain, d) of the right (R) and left (L) hemispheres; by hemisphere within each tissue layer, top row; by tissue layer within the right and left hemispheres, bottom row. **(B)** Torsion (top row; –100 to 100 mm^–1^; × 10^–3^) and curvature (bottom row; 0 to 5 mm^–1^; × 10^–2^) values by hemisphere (R,L) according to the three tissue layers (g,w,d): interhemispheric differences, superior handles; inter-tissue-layer differences, inferior handles; purple handles, torsion, and orange handles, curvature.

The comparison of values of deformation parameters by tissue layer and by hemisphere showed tangible differences (random-effects model), and more markedly for the directional electrodes. The complete data are shown in Figure 10 and Supplementary Table 1. The comparison of x, y, and z deformations between the right and left hemispheres showed a first-hemisphere-brain-shift effect for the x deformation of directional electrodes, whatever the tissue layer.

The values of intraclass coefficient of parameters per type of electrodes, hemispheres, and tissue layers (Table 2) showed that the ICC were mostly high and moderate for x, y, and z deformations, whereas the ICC were, respectively, always and mostly low for torsion and curvature.

**TABLE 2:** Intraclass coefficient (ICC) values (high, green; moderate, blue; gray, low; see text for details) of the deformation parameters according to the type of electrode (non-directional, directional) and the hemisphere (right, left).

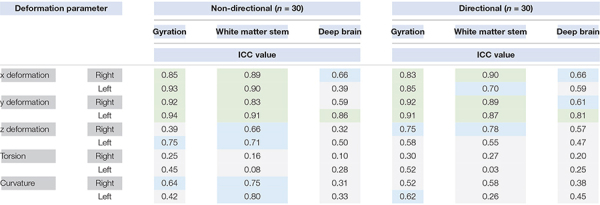

We observed differences of x, y, and z deformations by tissue layers except between the WMS and the DB for x-right and z-right of non-directional electrodes, and between the gyration and the DB layers for y-left of directional electrodes. The torsion values were different by hemisphere except in the WMS for the non-directional electrodes and in the DB layer for the directional electrodes; coarsely, the torsion was higher, in the right hemisphere with the non-directional electrodes and in the left hemisphere with the directional electrodes. We only observed in-between layer differences of torsion for the directional electrodes. The differences of curvature values by hemispheres were inverted relatively to the torsion, i.e., higher in the left hemisphere for the non-directional and in the right hemisphere for the directional (not in the DB). The in-between layer differences of curvature were observed for the two types of electrodes (except between the gyration and the DB layer in the left hemisphere).

In the deep brain, where the electrodes were terminated, the comparison of directional versus non-directional electrodes showed differences of deformation parameters. Thus, we observed the following for the non-directional electrodes: x deformation toward the midline in the right and left hemispheres; y deformation toward posterior in the right hemisphere; z deformation less deep in the left hemisphere; higher curvature values in the right hemisphere (Supplementary Table 1). Visually, the rigid parts of electrodes showed limited deformations.

## Discussion

We aimed to characterize the surgical-related inaccuracy, the brain shift, and the electrode deformation that could explain the observed mismatch between the position of the intended-to-place electrode and the final position of the electrode immediately after implantation. Two kinds of electrodes, non-directional and directional, which should have different rigidities according to the materials and the design, were studied. Our analysis of the deformations of electrodes and brain shift index showed tangible, although limited, deformations that provide insights on the intracranial, brain, and electrode mechanics. Broadly, the surgical-related inaccuracy was well controlled, on average less than 1.5 mm, in the overall population of patients (*n* = 30), irrespective of the kinds of electrodes, non-directional or directional. This value is rather low, bearing in mind that it is the result of several surgical issues related to the tools, the reading of rules, the progression in the brain, and the securitization of electrodes. The distance vectors (surgical-related inaccuracy) compare favorably with those observed with much more complex intraoperative surgeries, such as intraoperative MRI guidance (Matias et al., 2018). In any case, this should not mask us the large variability of reported surgical-related inaccuracy (Kluger et al., 2011; Engel et al., 2018; Roth et al., 2018; Sheehy et al., 2019). In our series, we also found that the left-hemisphere surgical-related inac+

curacy was slightly superior for the directional electrodes, 1.5 ± 0.5 mm, than for the non-directional electrodes, 1.06 ± 0.5 mm. This could be due to mechanical properties of the electrodes and the first-electrode implantation in the left hemisphere. Some elements point to the higher rigidity of the directional electrode: the percentage of the rigid part is longer, 31.77 ± 1.7 mm (versus 11.1 ± 0.6 mm); the polyurethane 55D is harder (versus 80A); and eight (versus four) electric wires go through the wall of the probe. Indeed, the intrinsic flexural stiffness of the electrodes, not provided by the manufacturers, should also play a role. This parameter is however complex because of the heterogeneity of the electrode notably along its length, such as the number of electric wires, the zone of welding, the crimped contacts, and the type of material of the tube probe. The specific impact of the distal rigid parts of the two types of electrodes should be further studied.

The specificities of deformations of the two electrode types by hemisphere brought other information: the non-directional electrodes seem more flexible as they bent toward the midline and posteriorly and had a higher curvature in the left hemisphere, whereas the directional electrodes bent laterally in the left hemisphere and posteriorly in the right hemisphere; both types of electrodes deformed to the depth in both hemispheres. As a reminder, the small brain shift observed in our series, through the brain-shift index, was objectively higher in most cases on the left where we systematically performed the first implantation, and the softer electrodes, non-directional, bent more easily following the brain displacement. The practical consequence is that we see that the brain shift can impact significantly the electrode trajectory, as already known (Zrinzo et al., 2009), and sometimes up to long-term (Contarino et al., 2013), but our findings illustrate that it is also true for very small volumes of extracerebral pneumocephalus. However, in our institution, we use the CFS-proof-oriented technique (dural puncher; 6-mm-diameter craniotomy) which minimizes the dura opening to the diameter of the electrode, i.e., about 1.27 mm, just enough to go through the hole, following a published method (Vassal et al., 2012). Moreover, the patient lays in strict recumbent position with the head flat, in the operating room and during imaging procedures (MRI and CT scan); this also minimizes the air penetration as the intracranial pressure does not drop (as compared to elevated head). Overall, a first-hemisphere-brain-shift effect coupled with an electrode-type effect therefore appears.

Our findings on the deformation parameters according to the tissue layers revealed features according to the type of electrodes. The non-directional electrodes showed, roughly, slightly less values of x, y, and z deformations, as if they less deformed the brain tissue, because of their relative softness, except in the deep brain. Besides in the deep brain, the non-directional electrodes bent toward the midline (right and left hemispheres), posterior (right hemisphere), and less deep (left hemisphere) and had higher curvature (left hemisphere). The dramatic changes in torsion and curvature values, Figure 9 showed that the white matter stem region sets a transitional zone, where most of the variations of mechanical modifications of the electrodes that cross the brain happen relatively abruptly. However, the important variability (ICC values) of torsion and curvature values limits this interpretation. The difference of x deformations between the right and left hemispheres was more frequently observed in the gyration layer irrespective of the electrode type. It is as if the gyration layer reflected somewhere a sort of mobility of the brain through a gyrus shifting, likely linked with the first-hemisphere-brain-shift effect (see above).

All other things being equal, the electrodes showed quasi-systematically a tissue layer effect in both hemispheres, which might reflect a regionalized coupling of the reciprocal mechanical relationships between the electrode and the layer. Yet this was not true for the z deformation in the right hemisphere between the WMS and the DB for the non-directional electrodes, and for the y deformation in the left hemisphere between the gyration and the DB for the directional electrodes. The mechanical stress state of the brain tissue, which was not explored in our study, could play a role. We only focused on the early static deformations resulting from the implantation.

Overall, it therefore appears that the type of electrodes interacts regionally across each tissue layer, and this interaction depends on the hemisphere, which in turn, following the abovementioned findings, depends on the first-hemisphere-brain-shift effect.

The brain is classically considered as a viscoelastic medium, in which regional differences exist, such as gray versus white matter, cortex versus thalamus or hippocampus, and anisotropy of white matter fiber orientation in the corona radiata and the corpus callosum (Hrapko et al., 2008; Jin et al., 2013; Moran et al., 2014; Finan et al., 2017; McCarty et al., 2019). As we have seen, clinically, it also seems to make sense to subdivide the brain into different mechanical regions or layers. Indeed, the mesencephalo-prosencephalic brain, i.e., the hemispheres, the midbrain, and the upper brainstem, which is relevant for the study of DBS surgery, should be segregated into three layers: the gyration layer, cortico-para-subcortical, where the gyrus and sulcus are developed; the intermediate white matter layer, which corresponded to the white mater stem surmounting the deep brain, which contains the well-known corona radiata, crossed by large white matter fascicles; and the deep brain layer, which corresponds to the region of deep-seated structures, a blended structure made of intermingled central gray nuclei and white matter fascicles targeted in functional surgery.

In conclusion, our findings could help in further development of invasive electrode design engineering, such as elastic probes (Ahmadi et al., 2018), or modification of mechanical properties of electrodes (surface, stiffness), enabling easy and reliable progression across tissue layers, as well as modification of surgical techniques and new tools such as simultaneous bilateral intracerebral implantation, highly cerebrospinal liquid-proof techniques, and robotized partitioned probes. One should bear in mind that the relatively high stiffness of the probe is an advantage, as it facilitates the progression along a linear trajectory, but it is otherwise if a rigid stylet is present, and conversely it can be again an advantage if the probe progresses without an internal system of rigidity. Our results also raise the veil on the mechanical inhomogeneity (state or properties), organized by layers, of the brain medium, which interacts in a complex manner with both the invasive electrodes and the intracranial environment. Further studies exploring the static and dynamic mechanics of both the brain and the electrodes should be useful, either experimentally or using finite element models. Our data, collected in clinical conditions, should add to the knowledge of biomechanical properties of the human brain.

## Data Availability Statement

The raw data supporting the conclusions of this article will be made available by the authors, without undue reservation.

## Ethics Statement

The studies involving human participants were reviewed and approved by Commission Nationale de l’Informatique et des Libertés, CNIL, agreement M200702. The patients/participants provided their written informed consent to participate in this study.

## Author Contributions

FC conceptualized, designed, analyzed, and wrote the first draft of the manuscript. LM analyzed and contributed substantially to the interpretation of data and wrote the first draft of the manuscript. BP analyzed and contributed substantially to the statistical analysis and wrote sections of the manuscript. JC, YE, and RC analyzed and wrote sections of the manuscript. AS analyzed the data curation and collection and wrote sections of the manuscript. DG contributed substantially to the interpretation and review of data. YL conceptualized and contributed substantially to the interpretation and review of data. BC analyzed the data curation and collection. YB and VS analyzed and reviewed notably material aspects. AM analyzed notably patients’ data and wrote sections of the manuscript. AW analyzed notably mathematical issues and designed and wrote sections of the manuscript. J-JL conceptualized, designed, analyzed, and wrote the first draft of the manuscript and prepared the figures. All authors contributed to the article and approved the submitted version.

## Conflict of Interest

The authors declare that the research was conducted in the absence of any commercial or financial relationships that could be construed as a potential conflict of interest.
