# Supplementary material for: Early Deformation of Deep Brain Stimulation Electrodes Following Surgical Implantation: Intracranial, Brain, and Electrode Mechanics
Source: Front Bioeng Biotechnol. 2021 Jun 11;9:657875. doi: 10.3389/fbioe.2021.657875 (PMC8226181; doi:10.3389/fbioe.2021.657875)
Supplement: Supplementary file 1 [file Table_1.DOCX]

**SUPPLEMENTARY TABLE S1**: Deformation parameters according to the tissue layers: G, gyration; WMS, white matter stem; DB, deep brain; vs, versus; R and L, right and left hemisphere; p value, random-effect model.

| deformation parameters | |  | non-directional (n=30) | | | | | |  | directional (n=30) | | | | | |  | directional vs non-directional; DB |
| --- | --- | --- | --- | --- | --- | --- | --- | --- | --- | --- | --- | --- | --- | --- | --- | --- | --- |
|  |  |  | gyration (G) | white matter stem (WMS) | deep brain (DB) | G vs WMS | G vs DB | WMS vs DB |  | G | WMS | DB | G vs WMS | G vs DB | WMS vs DB |  |  |
|  |  |  | *median interquartile range [ IRQ25; IRQ75]* | | | *p value* | | |  | *median interquartile range [ IRQ25; IRQ75]* | | | *p value* | | |  | *p value* |
|  |  |  |  |  |  |  |  |  |  |  |  |  |  |  |  |  |  |
| x deformation (mm) | R |  | 0.18 [-0.02; 0.30] | 0.21 [0.02; 0.34] | 0.22 [0.03; 0.33] | 0.003 | 0.014 | 0.12 |  | 0.17 [0.04; 0.29] | 0.19 [0.06; 0.31] | 0.04 [-0.10; 0.18] | 0.03 | <0.001 | <0.001 |  | 0.0013 |
|  | L |  | 0.19 [-0.36; 0.38] | 0.28 [0.02; 0.44] | 0.22 [0.09; 0.37] | <0.001 | <0.001 | <0.001 |  | -0.2 [-0.37; -0.10] | -0.13 [-0.25; -0.01] | -0.05 [-0.21; 0.007] | <0.001 | <0.001 | < 0.001 |  | <0.001 |
|  | *R vs L, p value* |  | <0.001 | 0.09 | 0.12 |  |  |  |  | <0.001 | <0.001 | <0.001 |  |  |  |  |  |
| y deformation (mm) | R |  | 0.20 [-0.12; 0.52] | 0.09 [-0.17; 0.28] | -0.10 [-0.21; 0.05] | <0.001 | <0.001 | <0.001 |  | 0.49 [0.30; 0.78] | 0.36 [0.24; 0.60] | 0.09 [-0.07; 0.27] | <0.001 | <0.001 | <0.001 |  | 0.05 |
|  | L |  | 0.34 [0.04; 0.58] | 0.16 [-0.02; 0.47] | 0.06 [-0.09; 0.19] | <0.001 | <0.001 | <0.001 |  | 0.01 [-0.18; 0.28] | 0.00 [-0.19; 0.15] | 0.05 [-0.18; 0.17] | <0.001 | <0.001 | 0.27 |  | 0.8 |
|  | *R vs L, p value* |  | <0.001 | 0.001 | <0.001 |  |  |  |  | <0.001 | <0.001 | <0.001 |  |  |  |  |  |
| z deformation (mm) | R |  | 0.10 [-0.21; 0.02] | -0.14 [-0.26; 0.03] | -0.17 [-0.34; 0.06] | 0.03 | <0.001 | 0.09 |  | 0.02 [-0.08; 0.32] | 0.08 [-0.16; 0.32] | 0.10 [-0.20; 0.24] | <0.001 | <0.001 | 0.001 |  | 0.09 |
|  | L |  | -0.04 [-0.20; 0.33] | -0.12 [-0.30; 0.04] | -0.18 [-0.30; 0.04] | <0.001 | <0.001 | <0.001 |  | 0.20 [-0.06; 0.44] | 0.09 [-0.16; 0.32] | 0.00 [-0.23; 0.22] | <0.001 | <0.001 | <0.001 |  | 0.028 |
|  | *R vs L, p value* |  | <0.001 | 0.72 | 0.12 |  |  |  |  | < 0.001 | 0.62 | 0.001 |  |  |  |  |  |
| torsion (mm^-1^ × 10^-3^) | R |  | 1.6 [-2.2; 6.7] | 16.5 [-22.7; 84.9] | 2.1 [-1.2; 6.4] | 0.07 | 0.88 | 0.08 |  | -2.5 [-10.6; 1.7] | -16.8 [-80.8; 20.4] | -3.0 [-14.4; 2.9] | 0.02 | 0.62 | 0.04 |  | 0.35 |
|  | L |  | -1.9 [-12.8; 1.5] | -34.4 [-110.0; 7.7] | -1.9 [-9.4; 0.7] | 0.36 | 0.86 | 0.26 |  | 3.0 [-1.9; 8.9] | 36.1 [-3.6; 108.3] | 3.1 [0.1; 10.2] | <0.001 | 0.98 | <0.001 |  | 0.06 |
|  | *R vs L, p value* |  | <0.001 | 0.21 | <0.001 |  |  |  |  | <0.001 | 0.004 | 0.29 |  |  |  |  |  |
| curvature (mm^-1^ × 10^-3^) | R |  | 2.5 [1.4; 3.6] | 1.0 [0.5; 1.5] | 3.0 [1.9; 4.5] | <0.001 | <0.001 | <0.001 |  | 2.6 [1.8; 3.8] | 0.8 [0.5; 1.2] | 2.3 [1.4; 3.3] | <0.001 | <0.001 | <0.001 |  | 0.04 |
|  | L |  | 2.7 [1.9; 4.3] | 1.2 [0.9; 2.2] | 3.0 [1.5; 4.6] | <0.001 | 0.48 | <0.001 |  | 2.3 [1.4; 3.4] | 0.7 [0.4; 1.0] | 2.2 [1.4; 3.4] | <0.001 | 0.97 | <0.001 |  | 0.06 |
|  | *R vs L, p value* |  | <0.001 | <0.001 | 0.04 |  |  |  |  | <0.001 | 0.003 | 0.29 |  |  |  |  |  |
